# Atlanta Academy of Medicine

**Published:** 1878-10

**Authors:** J. G. Westmoreland

**Affiliations:** Reporter


### ATLANTA ACADEMY OF MEDICINE.

J. G. WESTMORELAND, M.D., Reporter.

Atlanta, Ga., Oct. 7, 1878.

Academy met, Dr. J. F. Alexander, President, in the chair.

Dr. J. G. Westmoreland reported a case of caruncle of the female meatus urinarius, which had been twice ineffectually removed by pinching it off with forceps. He proposes to clip it perfectly with scissors or the knife, and cauterize well the surface from which it is removed, with the hope of preventing a return of the tumor. This growth gives much suffering and inconvenience to females, and sometimes leads to very annoying reflex nervous symptoms.

Dr. Calhoun asked information in regard to the structure of such tumors, to which Dr. W. replied that they resembled very much the ordinary polypus, being very vascular and easily lacerated.

Dr. Scott thought the cauterization of the part from which the caruncle is removed, with nitrate of silver, is likely to lead to granulations, and is therefore not so desirable, for this purpose, as the fuming nitric acid or the acid nitrate of mercury, which lead to better results.

Dr. Wilson said he had removed a caruncle with scissors and cauterized with nitrate of silver, which resulted in apparent permanent cure up to the time he lost sight of the case some months after the operation.

Dr. J. F. Alexander mentioned a case in which several carunculous tumors were found scattered along in different portions of the urethra, giving very serious nervous symptoms. Their removal gave great relief to the patient, but owing to the failure to cauterize the points of attachment, the growths have returned, and with them the unpleasant nervous state. He proposes soon to remove them more effectually.

Dr. H. L. Wilson reported a case of stricture in the male urethra, in which uræmic poisoning followed an operation with Civeale's urethrotome. Three strictures were found to exist—one an inch or two from the meatus, one near the membranous portion and the other intermediate. The operation was performed, and the patient did well for several days, a bougie of considerable size being introduced daily. Finally the patient became negligent of reporting, and failed to pass the bougie to the bladder, and urine failed to pass, either from suppression or retention in the bladder, and with this failure to pass the urine, symptoms of uræmia made their appearance, such as stupor and delirium. His condition became decidedly alarming, but gave way to an abundant secretion and free discharge of urine.

---

Atlanta, October 14, 1878.

The Academy met; Dr. J. F. Alexander, President, in the chair.

Dr. J. M. Johnson reported a case of dysmenorrhœa, with pseudo-membrane, which he said had been under the care of Dr. Battey, and was considered incurable. The case was turned over to Dr. Johnson while in an emaciated condition. In two or three months the patient improved in strength; in six months the false membrane ceased to pass at the menstrual period, as was her habit; in nine months was married, and in twenty-three months bore a healthy child.

The treatment consisted of the internal use of bichloride of mercury in the form of tincture of anticrida, and muriate of ammonia in the dose of fifteen grains three times a day. The triple phosphites also composed part of the internal treatment. The local treatment consisted principally of the injection into the vagina of the muriated tincture of iron in the strength of one part to two of water. His opinion is, that the tincture is taken into the uterus from the vagina by a kind of suction, and makes its impression upon the internal surface, so as to change the condition of the intra-uterine surface.

On being interrogated in regard to the nature of the false membrane discharged at each monthly period, Dr. Johnson said, various authors have given opinions on the subject. Some conclude that it is nothing more nor less than mucous membrane, others that it is similar to the decidua, and others that it is the chorion. He is inclined to agree with those advocating the latter opinion.

Dr. Rauschenberg thinks the membrane discharged is nothing more nor less than a proliferation of the epithelial portion of the mucuous membrane, and that this opinion is entertained by the best pathologists.

Dr. Scott agreed with Dr. Rauschenberg, that the membrane discharged is epithelial, and resembles the exfoliation of the external cuticle, and is analagous to the false membrane in croup. He can explain the treatment with mercury instituted by Dr. Johnson on this hypothesis: It is known that this remedy is effectual in membranous croup, and hence, for the pseudo-membrane in question.

Dr. Rauschenberg called attention to a case of leucæmia—a specimen of blood from whom was presented under the microscope, but owing to the imperfect light, he could not exhibit the appearance to-night, but hopes to be able to do so at the next meeting. The blood is found to contain only five per cent. of red corpuscles, compared with the white.

Dr. Calhoun reported a case of epithelial cancer between the lid and ball of the eye. The patient is thirty-five years of age, and has had the disease for eighteen years. The disease extends now from the inner canthus along the upper lid, and also affecting the under lid. The ball was somewhat affected with the disease, and was completely concealed by the diseased and enlarged granulated lids. The rapid growth of the granulations is very marked, and he inquired of members having experience, what significance should be attached to this circumstance.

The treatment consists of the removal of the lids and ball entire.

Dr. W. F. Westmoreland reported a case of encephaloid cancer of the humerus, requiring amputation at the shoulder joint. The operation was performed after the use of Esmark's tourniquet, or the rubber bands. The arm was so completely deprived of blood that not a drop was lost in the operation. Even the knife, with which the operation was made, did not have the stain of blood upon it.

The specimen was exhibited, and was found to have commenced at the elbow joint, extending so near to the shoulder joint that the amputation below the joint was not considered likely to be effectual. The disease commenced eight or ten months since without any known cause.

The patient was anæmic from having resided in a malarious district, hence the care exercised to prevent any loss of blood. The operation being made under anæsthesia, no shock was communicated to the nervous system; and the freedom from loss of blood, leaving the circulation perfect, the patient was put to bed in an undisturbed state.

---

ATLANTA, Ga., Oct. 26, 1878.

Academy met; Dr. Todd, Vice-President, presiding.

On call for cases, Dr. Baird mentioned two of diphtheria occurring in his practice, which were especially prominent from the extensive membrane upon the posterior wall of the pharynx; other symptoms were those peculiar to ordinary cases of the disease. Used as a local application, concentrated chlorate of potash in solution, with muriated tincture of iron. A few days after seeing patients, he commenced with the use of the tincture of iron internally. Patients pulse being 130, he had used veratrum.

Dr. Scott recommended very highly a preparation of lime as a local applicant—using either warm lime-water through atomizer, or to be penciled on affected surfaces, or, as he would prefer, the fumes of lime while undergoing process of slaking. He stated that the fumes, especially, were quite soothing to the diseased surfaces, and besides possessed the property of dissolving the diphtheritic membranes, and disinfecting them so as to obviate poisoning of blood from their absorption. Lime also seemed to have the power of destroying the vegetable parasities, which latter, in the view of many pathologists, were the cause of the disease.

Dr. W. F. Westmoreland had known lime water inhalations to have a happy effect in cases of croup, but had had no experience with it in diphtheria.

Dr. Scott replied to an enquiry of Dr. Cumming, that his method was to place quick lime in a cup with a little water, and having formed a cone with a towel so as to envelop the cup and mouth and nose of the patient so as to cause the fumes to be readily inhaled; thought there was undoubtedly inhaled, in addition to the steam from water, the essential principles of the lime.

Dr. Baird, in reply to Dr. Cumming, as to his reasons for not giving tr. iron at commencement of disease, stated that he first endeavored to control frequency of heart's action by using cardiac sedatives, and then afterwards began using tr. iron.

Dr. W. F. Westmoreland, in reply to Dr. Todd, commences with the use of tr. iron immediately after clearing the bowels with a mild dose of calomel, both in diphtheria and erysipelas; thought cardiac sedatives were not indicated, unless excessive action was destroying the patient—for instance the pulse from 135 to 140 in the minute.

Dr. Todd mentioned having read a report by Dr. DaCosta of several cases of erysipelas occurring in the Pennsylvania Hospital, and from the long duration of these cases he had become impressed with the idea that they would have progressed more favorably had the use of tr. iron been preceded by some preparation of mercury—thought clearing of bowels had a happy effect as a forerunner in the treatment.

Dr. W. F. Westmoreland remarked that erysipelas occurred under various forms, and these were of variable duration; the phlegmonous or cellular form could not be arrested short of suppuration, and necessarily lasted weeks.

Dr. Todd inquired if Dr. W. F. Westmoreland placed any especial stress on albumen in urine?

Dr. W. F. Westmoreland does not lay much stress on albumen; where pus comes intimately in contact with vessels and there is no barrier to its absorption, as in phlegmonous variety, albumen was to be found in urine.

Dr. Baird recognizes the fact that where there is tendency to death by asthenia, cardiac sedatives were to be guardedly administered; but thinks that where the force or frequency of heart's action is such as to tend to rapidly wear out patient and produce alarming brain symptoms, preperations such as veratrum were surely indicated. This was the condition present in his cases above reported,—pulse being over 130 per minute.

Dr. W. F. Westmoreland presented tumor of parotid, of malignant character, which he had removed from a patient on Saturday last.

Some eight months previous, patient had presented himself with small tumor under ear, evidently growing from parotid gland; had at that time advised its removal, which could have been readily done, but was unable to obtain consent of patient.

This tumor had been in existence some ten or fifteen years, but had recently grown very rapidly indicating somewhat a malignant nature.

Being unable to dissuade patient from operation he finally yielded and removed tumor—size of fist, consisting of entire parotid gland.

This operation, when he was a student, was considered an impossibility—Dr. Gibson saying it could n't be done without ligating the common carotid or death of patient; soon afterwards Pancoast successfully executed it and presented specimen to Dr. Gibson demonstrating its feasibility.

In case under consideration, after exposing tumor he obviated hemorrhage from arterioles by a process of tearing or hulling it out from its attachments, using finger-nail or a bone-knife; didn't ligate any vessels until tumor had been removed and recurrent or back-hemorrhage ensued.

Before beginning operation he had been unable to detect any pulsation in primitive carotid of affected side, and imagined it might be included in centre of tumor; this was afterwards found not to be the case; still even then he was unable by careful search to detect pulsation of the artery, and at the time thought he saw rudiments of an obliterated carotid. His present opinion is that this was an erroneous supposition, because of absence of all symptoms on the part of jugular vein and par vagum, which would necessarily have been more or less prominent during an action sufficient to have caused artery's obliteration. Waited an hour for reaction and ligated about twenty arterioles; profuse hemorrhage returning he used persulphate of iron and closed the wound, allowing however, no sponge to remain in wound on account of its tendency to rapid decomposition. Patient on morning following operation called for his breakfast at 7 o'clock, and there has been no unpleasant symptom up to date.

Dr. Connally inquired if Dr. Westmoreland regarded it as a malignant tumor.

Dr. Westmoreland said it was of malignant character; he had known tumors to exist for thirty years and finally all at once take on a malignant action.

Dr. Baird reported a case of tetanus in boy seven years old; ten days previous found patient with pain between shoulders, stiffness of the muscles and one side of face, giving rise to peculiar condition known as risus sardonicus, squinting of left eye, left leg stiff, screaming at night, and intense pain in abdomen, with spasmodic contractions of diaphragm.

Surmising worms, gave calomel and santonin—two gr. calomel three gr. santonin, repeated twice, and followed by castor oil, which caused several worms of the genus *ascarides vermicularis* to be passed; still the symptoms increased, until the least touch would throw him into tetanic spasms; indeed, about the sixth day from attack he would have as many as fifty spasms nightly. And then began with hydrate of chloral and pot. brom. two to seven grs. combined, gradually increasing doses, and patient has continued improving up to present time—the frequency of spasms being reduced nearly one-half.

Dr. W. F. Westmoreland considers the case to be idiopathic tetanus, so called, though this was a misnomer and only an expression of our ignorance, and thinks boy will recover. If such a case can be controlled by anti-spasmodics, he should say it was not tetanus proper; has known in his own practice only two genuine cases to get well. Thinks chloroform may be given with impunity.

Dr. Scott remarked that he had given especial attention to the subject of traumatic tetanus, and had written an article based upon several *post mortems* held upon those dying in Langenbeck's clinic from that affection. These and other reported cases there, showed that in most cases the principal nerves leading from the injured parts were softened and swollen, together with more or less hyperæmia or an extravasation of blood within the neurilemma; these changes being the more marked and extending the higher up towards spinal cord the longer the affection had continued; in some cases the entire nerve up to its entrance into spinal cord was found affected.

Based upon these discoveries, he had in the above article recommended section, and, if deemed necessary, excision of a portion of the nerve trunk at *a point remote from the injury*, thus enabling us to cut off the central nervous system entirely from all irritating causes. He thought this should be done early, before the brain and spinal cord had become altered and excited to that point where peripheral irritation was no longer necessary to keep up spasms; stated the operation was easily executed, and without any danger to the life of the patient, and far preferable to amputation of the parts, as recommended by Larrey, Petit, and others—Petit having said amputation was a sure cure if done at once on the outbreak of the affection; could not believe with Dr. Westmoreland, that chloroform could be given with impunity in this affection; knew of a case where it was administered during the operation for removal of foreign body with great relief to spasms, but on a second spasm ensuing it was again administered, and the patient died instantly; said it was well known that patients either died from exhaustion or from *spasm of the glottis*, and therefore chloroform was too risky a remedy; spoke highly of tracheotomy as a *dernier resort*.

Dr. W. F. Westmoreland has in some cases divided the nerves and soft parts surrounding the injury, and in one case saw benefit ensue; thinks death is caused by a falling back of epiglottis, rather than spasm of glottis, and recommends grasping of the tongue with forceps and pulling it forward. If not sufficient to relieve the symptoms, should perform tracheotomy. A year ago this little boy was stuck with a needle, which had as yet not been removed.

Dr. Lowe reminded the Academy of a case where a woman swallowed a paper of needles, some of which afterwards made their appearance under integument of abdomen.

Academy adjourned.

